# Lenvatinib plus drug-eluting bead transarterial chemoembolization with/without hepatic arterial infusion chemotherapy for hepatocellular carcinoma larger than 7 cm with major portal vein tumor thrombosis: a multicenter retrospective cohort study

**DOI:** 10.1097/JS9.0000000000001819

**Published:** 2024-06-13

**Authors:** Mingyue Cai, Licong Liang, Jian Zhang, Nianping Chen, Wensou Huang, Yongjian Guo, Xiaotao Hong, Liteng Lin, Yaohong Liu, Cao Dan, Haihui Deng, Xiaoguang Liu, Jingwen Zhou, Ye Chen, Huanwei Chen, Kangshun Zhu

**Affiliations:** a Department of Minimally Invasive Interventional Radiology, The Second Affiliated Hospital of Guangzhou Medical University; b Department of General Surgery, Guangzhou Development District Hospital, Guangzhou; c Department of Interventional Medicine, Zhongshan City People’s Hospital, Zhongshan; d Department of Hepatobiliary and Pancreatic Surgery, Affiliate Hospital of Guangdong Medical University, Zhanjiang; e Department II of Oncology, Jieyang People’s Hospital, Jieyang; f Department of Vascular and Interventional Radiology, Huizhou Municipal Central Hospital, Huizhou; g Department of Interventional Radiology, Shenzhen Traditional Chinese Medicine Hospital, Shenzhen; h Department of Hepatopancreatic Surgery, the First People’s Hospital of Foshan, Foshan, China

**Keywords:** combination therapy, hepatic arterial infusion chemotherapy, hepatocellular carcinoma, lenvatinib, portal vein tumor thrombosis, transarterial chemoembolization

## Abstract

**Background:**

The management of hepatocellular carcinoma (HCC) with high tumor burden and major portal vein tumor thrombosis (PVTT) remains a great challenge. The authors aimed to investigate the efficacy and safety of lenvatinib plus drug-eluting bead transarterial chemoembolization (DEB-TACE) and hepatic arterial infusion chemotherapy (HAIC) with oxaliplatin, fluorouracil and leucovorin (Len+DEB-TACE+HAIC) versus lenvatinib plus DEB-TACE (Len+DEB-TACE) for HCC greater than 7.0 cm accompanied with major PVTT.

**Materials and methods:**

This multicenter retrospective cohort study evaluated consecutive patients with HCC (> 7.0 cm) and major PVTT who received Len+DEB-TACE+HAIC (Len+DEB-TACE+HAIC group) or Len+DEB-TACE (Len+DEB-TACE group) between July 2019 and June 2021 from eight institutions in China. Objective response rate (ORR), time to progression (TTP), overall survival (OS), and treatment-related adverse events (TRAEs) were compared between the two groups by propensity score matching (PSM).

**Results:**

A total of 205 patients were included. After PSM, 85-paired patients remained in the study cohorts. Patients in the Len+DEB-TACE+HAIC group had higher ORR (61.2% vs. 34.1%, *P* < 0.001), longer TTP (median, 9.8 vs. 5.9 months, *P* < 0.001), and prolonged OS (median, 16.7 vs. 12.5 months, *P* < 0.001) than those in the Len+DEB-TACE group. The ORR and TTP of both intrahepatic tumor (ORR: 64.7% vs. 36.5%, *P* < 0.001; median TTP: 10.7 vs. 7.0 months, *P* < 0.001) and PVTT (ORR: 74.1% vs. 47.1%, *P* < 0.001; median TTP: 17.4 vs. 7.6 months, *P* < 0.001) were better in the Len+DEB-TACE+HAIC group than the Len+DEB-TACE group. The frequency of grade 3-4 TRAEs in the Len+DEB-TACE+HAIC group were comparable to those in the Len+DEB-TACE group (38.8% vs. 34.1%, *P* = 0.524).

**Conclusion:**

The addition of HAIC to Len+DEB-TACE significantly improved ORR, TTP, and OS over Len+DEB-TACE with an acceptable safety profile for large HCC with major PVTT.

## Introduction

Highlights

- Lenvatinib plus drug-eluting bead transarterial chemoembolization (DEB-TACE) and hepatic arterial infusion chemotherapy (HAIC) significantly improved the overall survival of patients with hepatocellular carcinoma (HCC) greater than 7.0 cm and major portal vein tumor thrombosis (PVTT), compared to lenvatinib plus DEB-TACE.
- The triple combination therapy led to better tumor control, with a higher objective response rate and longer time to progression for overall tumor, intrahepatic tumor, and PVTT.
- The addition of HAIC to lenvatinib plus DEB-TACE was tolerable and more effective, suggesting that this triple combination might represent a better treatment option for patients with large HCC and major PVTT.

Hepatocellular carcinoma (HCC) is a common cancer and the third leading cause of cancer-related death worldwide^1^. This aggressive cancer is prone to invade the portal vein system, resulting in portal vein tumor thrombosis (PVTT)^2,3^. Approximately 13–45% of HCC patients are accompanied with PVTT at initial diagnosis^4^. Especially in those with large tumors (>5 cm), the incidence of PVTT is up to 58.5%^5^. Notably, the extent of PVTT is closely associated with patient survival^3,5^. The prognosis becomes extremely poor when the first-order branch or the main trunk of the portal vein is involved (known as major PVTT)^6–8^. Given that both a large tumor burden and the presence of major PVTT are indicative of more advanced disease, treatment difficulties and worse outcomes^3,5,8–10^, the management of HCC patients with these combined conditions is even more challenging.

Lenvatinib has been recommended as one of the first-line treatment options for advanced HCC since the REFLECT trial demonstrated that lenvatinib was non-inferior to sorafenib in overall survival (OS)^5,10–13^. However, the efficacy of lenvatinib monotherapy is modest, with a median OS of 13.6 months^14,15^. Recently, the phase III LAUNCH trial showed that, compared with lenvatinib alone, lenvatinib plus transarterial chemoembolization (TACE) yielded significantly better OS, higher objective response rate (ORR), and longer progression-free survival in patients with advanced HCC^16^. These results implied that adding an efficacious locoregional treatment, such as TACE, to the systemic treatment with lenvatinib could sharply reduce intrahepatic tumor burden for better tumor control and thereby improve survival even in patients with vascular invasion and/or extrahepatic spread. However, for large HCC, the efficacy of TACE was limited^9,17,18^. Especially for very large HCC (> 7.0 cm), the ORR of TACE per modified Response Evaluation Criteria in Solid Tumors (mRECIST) was only 18.4–32.7%^17,19^. Therefore, in patients with a high tumor burden, a more potent locoregional combination therapy for controlling intrahepatic tumors is urgently needed.

In recent years, increasing evidence has indicated that hepatic arterial infusion chemotherapy (HAIC) with oxaliplatin, fluorouracil and leucovorin had significant therapeutic effects on unresectable/advanced HCC^17,20–22^. Our previous study has revealed that drug-eluting bead (DEB) TACE (DEB-TACE) plus HAIC (DEB-TACE+HAIC) could provide better tumor response and survival over DEB-TACE alone for large or huge HCC, especially those with non-smooth margin or macrovascular invasion^9^. These results suggested that DEB-TACE+HAIC might be superior to DEB-TACE alone in controlling large HCC with macrovascular invasion. Accordingly, lenvatinib plus DEB-TACE+HAIC (Len+DEB-TACE+HAIC) might provide favorable clinical benefits in HCC patients with high tumor burden and PVTT. Hence, we conducted this multicenter retrospective study to evaluate the efficacy and safety of Len+DEB-TACE+HAIC versus lenvatinib plus DEB-TACE (Len+DEB-TACE) for HCC greater than 7.0 cm accompanied with major PVTT.

## Methods

### Study design and patient selection

This multicenter retrospective cohort study was approved by the local institutional review board and registered at ClinicalTrials.gov. The requirement of informed consent was waived. This work was reported in accordance with the STROCSS criteria^23^ (Supplemental Digital Content 1, http://links.lww.com/JS9/C743).

This study was conducted in patients with HCC larger than 7.0 cm and major PVTT treated with Len+DEB-TACE+HAIC (Len+DEB-TACE+HAIC group) or Len+DEB-TACE (Len+DEB-TACE group) at eight institutions in China (The Second Affiliated Hospital of Guangzhou Medical University, Zhongshan City People's Hospital, Affiliate Hospital of Guangdong Medical University, Jieyang People's Hospital, Huizhou Municipal Central Hospital, Guangzhou Development District Hospital, Shenzhen Traditional Chinese Medicine Hospital, and The First People’s Hospital of Foshan) from July 2019 to June 2021. The inclusion criteria were as follows: (1) age between 18 and 75 years; (2) a confirmed diagnosis of HCC^24,25^; (3) the largest intrahepatic lesion greater than 7.0 cm; (4) presence of PVTT in the first-order branch or main trunk on imaging; (5) Eastern Cooperative Oncology Group performance status (ECOG PS) less than or equal to 1; (6) Child-Pugh class A/B; and (7) adequate hematologic and organ function, with leukocyte count greater than or equal to 3.0 × 10^9^/l, neutrophil count greater than or equal to 1.5 × 10^9^/l, platelet count greater than or equal to 75 × 10^9^/l, hemoglobin greater than or equal to 85 g/l, alanine transaminase and aspartate transaminase less than or equal to 5 × upper limit of the normal, creatinine clearance rate less than or equal to 1.5 × upper limit of the normal, and prothrombin time prolongation less than 4 seconds. The exclusion criteria were: (1) incomplete medical records; (2) central nervous system involvement; (3) previous treatment with transarterial embolization, TACE, HAIC, radiotherapy, or systemic therapy; (4) history of malignancies other than HCC; (5) history of organ transplantation; (6) severe cardiac, pulmonary, or renal dysfunction.

### DEB-TACE and HAIC procedures

All patients received standardized TACE^24,26^ with DEBs (CalliSpheres, Hengrui Medical; DC Bead, Biocompatibles), which was performed by physicians with more than 10 years of experience in interventional radiology (detailed in Supplementary Materials, Supplemental Digital Content 2, http://links.lww.com/JS9/C744). In order to reduce the risk of complications, the embolization endpoint with blood stasis of the tumor-feeding arteries was not achieved in a single treatment but in 2–3 DEB-TACE sessions^9,27^. If the tumors had extrahepatic feeding arteries, these arteries would be chemoembolized first. In the case of arterioportal shunting, the shunts were embolized with polyvinyl alcohol particles before injection of DEBs.

For HAIC, the microcatheter was not removed but reserved in the main tumor-feeding hepatic artery after chemoembolization^9^. Afterwards, the patients were transferred to the ward for drug infusion via the microcatheter: oxaliplatin, 85 mg/m^2^ for 2 h; leucovorin, 400 mg/m^2^ for 2 h; and flurouracil, 400 mg/m^2^ bolus and 2400 mg/m^2^ for 46 h.

DEB-TACE or DEB-TACE+HAIC was repeated for viable tumors demonstrated by follow-up imaging in patients without worsening liver function or contraindications^24^. In the case of clinical or functional deterioration, DEB-TACE or HAIC could be delayed and dose reduction of chemotherapy agents for HAIC was allowed^28,29^. In the Len+DEB-TACE+HAIC group, DEB-TACE or HAIC could be performed alone when the other one was discontinued due to adverse events (AEs) or technical difficulties. In both groups, the transarterial treatment was discontinued if objective response was not achieved after 2–3 consecutive treatment sessions.

### Lenvatinib administration

Lenvatinib (Eisai) was orally administered at a dose of 12 mg/day (bodyweight ≥ 60 kg) or 8 mg/day (bodyweight < 60 kg). It was initiated within 7 days after the first DEB-TACE or DEB-TACE+HAIC and continued until unacceptable toxicity or disease progression occurred. The interruption and dose reduction of lenvatinib was allowed and depended on the presence and severity of toxicities according to the package insert.

### Follow-up and assessments

The patients were followed up about 4 weeks after each DEB-TACE or DEB-TACE+HAIC and every 4-8 weeks thereafter until death or the end of the study (31 December 2022). Each follow-up assessment included detail history, physical examination, laboratory tests (complete blood cell count, liver function, creatinine, coagulation parameters and α-fetoprotein), contrast-enhanced abdominal computed tomography (CT) or magnetic resonance imaging, chest CT, and other examination if clinically indicated.

Tumor response was evaluated according to mRECIST by two independent radiologists at each participating center. If there was disagreement, the final decision was made by another senior radiologist. The extent of PVTT was classified per Japanese Vp classification^4,5^. PVTT responses were categorized by using a modified standard^30^, which was described in the Supplementary Materials (Supplemental Digital Content 2, http://links.lww.com/JS9/C744). The safety of treatment was assessed by monitoring treatment-related AEs (TRAEs) per Common Terminology Criteria for Adverse Events version 5.0.

### Outcomes

The differences in tumor response, time to progression (TTP), OS, and TRAEs were compared between the two groups. ORR was defined as the percentage of patients with complete or partial response. Disease control rate (DCR) was defined as the percentage of patients with complete or partial response, or stable disease. TTP was defined as the time from treatment initiation to the first occurrence of disease progression. OS was defined as the time from treatment initiation until death from any reason.

### Statistical analyses

To reduce potential selection bias and confounding between the two groups, a 1:1 propensity score matching (PSM) analysis was performed using nearest-neighbor method and a caliper width of 0.2 standard deviation without replacement^31^. Propensity scores were generated using a logistic regression model with variables of age, sex (male/female), hepatitis B surface antigen (HBsAg; positive/negative), ECOG PS (1/0), Child-Pugh class (B/A), albumin-bilirubin (ALBI) grade (2/1), α-fetoprotein, largest tumor size, number of tumors (> 3/≤ 3), tumor distribution (bilobar/unilobar), PVTT extent (Vp4/Vp3) and extrahepatic spread (present/absent). The standardized mean difference was used to examine the covariate balance between groups after matching (Supplementary Figure 1, Supplemental Digital Content 2, http://links.lww.com/JS9/C744). Additionally, sensitivity analyses were carried out by using different matching methods and factors for assessing the robustness of the PSM analysis (detailed in Supplementary Materials, Supplemental Digital Content 2, http://links.lww.com/JS9/C744).

Continuous variables were expressed as mean ± standard deviation or median with interquartile range (IQR), as appropriate, and compared using Student’s *t*-test or Mann–Whitney U test. Categorical variables were expressed as frequency (percentage) and compared using χ^2^ test or Fisher’s exact test, as appropriate. TTP and OS curves were generated by Kaplan–Meier method and compared by log-rank test. The independent effect of treatment modality on TTP and OS were confirmed by Cox regression analyses. Variables with *P* less than 0.100 in the univariate analysis were entered into the multivariate analysis. Subgroup analyses comparing TTP and OS between two groups were performed through Cox regression analyses. All statistical analyses were performed using R (version 4.3.1; R Foundation for Statistical Computing, https://www.r-project.org/) and SPSS Statistics (version 26; IBM). A two-tailed *P* less than 0.05 was considered statistically significant.

## Results

### Patient characteristics

**Figure 1:**
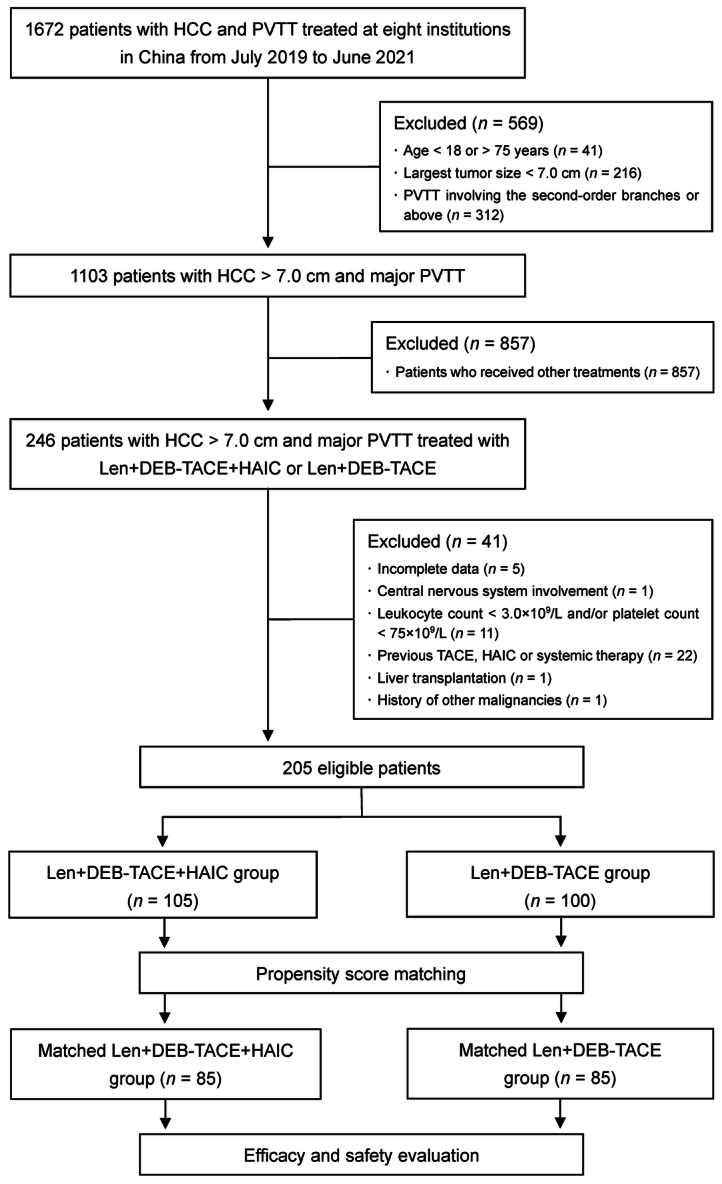
Flowchart of the study. HAIC, hepatic arterial infusion chemotherapy; HCC, hepatocellular carcinoma; Len+DEB-TACE, lenvatinib plus drug-eluting bead transarterial chemoembolization; Len+DEB-TACE+HAIC, lenvatinib plus drug-eluting bead transarterial chemoembolization and hepatic arterial infusion chemotherapy; PVTT, portal vein tumor thrombosis; TACE, transarterial chemoembolization.

A total of 205 patients were included in this study (Fig. 1), of whom 105 received Len+DEB-TACE+HAIC and 100 received Len+DEB-TACE. Before PSM, there was a significant difference in PVTT extent between two groups (*P* = 0.030). After matching, 85-paired patients with well-balanced baseline characteristics remained in the study cohorts (Table 1; Supplementary Figure 1, Supplemental Digital Content 2, http://links.lww.com/JS9/C744). The mean age was 52.7 ± 11.7 years, and 78 patients (91.8%) were male in the Len+DEB-TACE+HAIC group, while the mean age was 52.6 ± 11.0 years, and 76 patients (89.4%) were male in the Len+DEB-TACE group. The median largest tumor size was 12.3 (IQR: 10.0–15.1) cm in the Len+DEB-TACE+HAIC group and 11.7 (IQR: 9.6–15.5) cm in the Len+DEB-TACE group. More than half of the patients in each group had intrahepatic tumor number greater than 3, and approximately half of the patients in each group had Vp4 PVTT.

**Table 1:** Baseline characteristics of the patients.

	Total cohorts			Matched cohorts		
Characteristic	Len+DEB-TACE+HAIC group (*n* = 105)	Len+DEB-TACE group (*n* = 100)	*P*	Len+DEB-TACE+HAIC group (*n* = 85)	Len+DEB-TACE group (*n =* 85)	*P*
Age (years)^a^	52.8 ± 11.4	53.0 ± 10.9	0.829	52.7 ± 11.7	52.6 ± 11.0	0.978
≤ 60	76 (72.4)	71 (71.0)	0.826	61 (71.8)	60 (70.6)	0.866
> 60	29 (27.6)	29 (29.0)		24 (28.2)	25 (29.4)	
Sex, *n* (%)						
Male	97 (92.4)	89 (89.0)	0.404	78 (91.8)	76 (89.4)	0.599
Female	8 (7.6)	11 (11.0)		7 (8.2)	9 (10.6)	
HBsAg, *n* (%)						
Positive	90 (85.7)	88 (88.0)	0.629	75 (88.2)	74 (87.1)	0.816
Negative	15 (14.3)	12 (12.0)		10 (11.8)	11 (12.9)	
ECOG PS, *n* (%)						
1	15 (14.3)	13 (13.0)	0.789	11 (12.9)	11 (12.9)	> 0.999
0	90 (85.7)	87 (87.0)		74 (87.1)	74 (87.1)	
Child-Pugh class, *n* (%)						
B	15 (14.3)	9 (9.0)	0.239	9 (10.6)	9 (10.6)	> 0.999
A	90 (85.7)	91 (91.0)		76 (89.4)	76 (89.4)	
ALBI grade, *n* (%)						
2	83 (79.0)	75 (75.0)	0.491	64 (75.3)	65 (76.5)	0.858
1	22 (21.0)	25 (25.0)		21 (24.7)	20 (23.5)	
α-Fetoprotein (μg/l)^b^	286.4 (7.6–12 512.0)	662.4 (23.4–17 608.8)	0.340	880.6 (16.4–28 847.2)	780.5 (21.4–18 685.9)	0.980
≥ 400	51 (48.6)	53 (53.0)	0.526	46 (54.1)	48 (56.5)	0.758
< 400	54 (51.4)	47 (47.0)		39 (45.9)	37 (43.5)	
Largest tumor size (cm)^b^	11.5 (9.4–13.6)	11.9 (9.7–15.4)	0.254	12.3 (10.0–15.1)	11.7 (9.6–15.5)	0.843
> 10.0	69 (65.7)	70 (70.0)	0.512	62 (72.9)	59 (69.4)	0.611
≤ 10.0	36 (34.3)	30 (30.0)		23 (27.1)	26 (30.6)	
Number of tumors, *n* (%)						
> 3	52 (49.5)	51 (51.0)	0.833	46 (54.1)	44 (51.8)	0.759
≤ 3	53 (50.5)	49 (49.0)		39 (45.9)	41 (48.2)	
Tumor distribution, *n* (%)						
Bilobar	58 (55.2)	62 (62.0)	0.326	52 (61.2)	53 (62.4)	0.875
Unilobar	47 (44.8)	38 (38.0)		33 (38.8)	32 (37.6)	
PVTT extent, *n* (%)						
Vp4	60 (57.1)	42 (42.0)	0.030	43 (50.6)	41 (48.2)	0.759
Vp3	45 (42.9)	58 (58.0)		42 (49.4)	44 (51.8)	
Extrahepatic spread, *n* (%)						
Present	23 (21.9)	19 (19.0)	0.607	18 (21.2)	17 (20.0)	0.850
Absent	82 (78.1)	81 (81.0)		67 (78.8)	68 (80.0)	

Unless otherwise indicated, data are numbers of patients, with percentages in parentheses.

^a^ Data are means ± standard deviations.

^b^ Data are medians, with interquartile ranges in parentheses.

ALBI, albumin-bilirubin; ECOG PS, Eastern Cooperative Oncology Group performance status; HBsAg, Hepatitis B surface antigen; Len+DEB-TACE, lenvatinib plus drug-eluting bead transarterial chemoembolization; Len+DEB-TACE+HAIC, lenvatinib plus drug-eluting bead transarterial chemoembolization and hepatic arterial infusion chemotherapy; PVTT, portal vein tumor thrombosis.

The mean follow-up was 17.0 ± 6.4 months in the Len+DEB-TACE+HAIC group and 13.2 ± 5.7 months in the Len+DEB-TACE group (*P* < 0.001). Patients in the Len+DEB-TACE+HAIC group underwent a total of 238 transarterial treatments, with a mean of 2.8 ± 0.9, while patients in the Len+DEB-TACE group underwent a total of 269 transarterial treatments, with a mean of 3.2 ± 1.0 (*P* = 0.015). The median duration of lenvatinib administration was 9.7 (IQR: 6.3–12.7) months in the Len+DEB-TACE+HAIC group and 5.7 (IQR: 3.1–8.0) months in the Len+DEB-TACE group (*P* < 0.001).

### Efficacy

**Figure 2:**
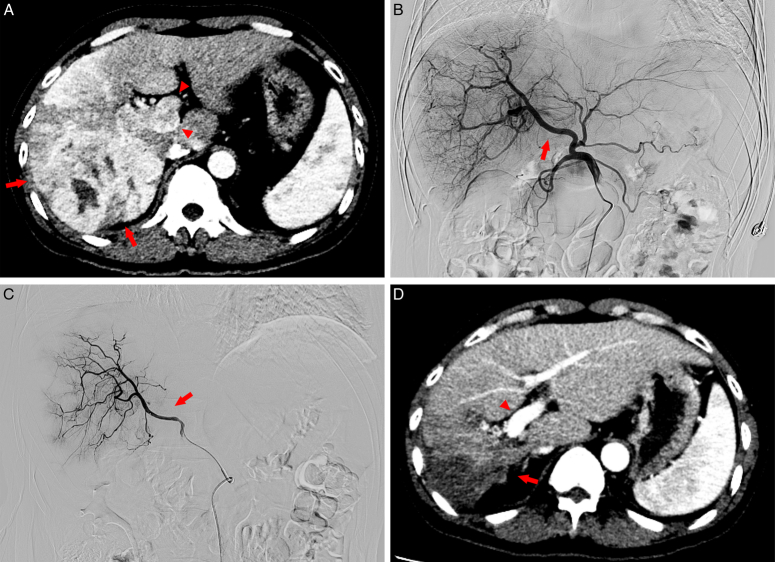
A 44-year-old man with huge hepatocellular carcinoma and Vp4 portal vein tumor thrombosis (PVTT) who received lenvatinib plus drug-eluting bead transarterial chemoembolization (DEB-TACE) and hepatic arterial infusion chemotherapy (HAIC). (A) Pretreatment contrast-enhanced computed tomography (CT) demonstrated an intrahepatic mass of 11.8 cm in the right lobe (arrows) with Vp4 PVTT (arrowheads). (B) Digital-subtraction angiography (DSA) before embolization showed the tumor-feeding vessels derived from right hepatic artery (arrow). (C) DSA after the first DEB-TACE showed that the tumor blush remained but significantly reduced, and the microcatheter was reserved at the right hepatic artery for HAIC (arrow); (D) Contrast-enhanced CT image obtained 2 months after three DEB-TACE and HAIC treatments demonstrated remarkable tumor shrinkage without enhancement inside the intrahepatic tumor and PVTT (arrow), with patency of the main trunk and left branches of portal vein (arrowheads).

Tumor responses of the two groups were shown in Table 2. The ORR of overall tumor (61.2% vs. 34.1%, *P* < 0.001), intrahepatic tumor (64.7% vs. 36.5%, *P* < 0.001) and PVTT (74.1% vs. 47.1%, *P* < 0.001) was significantly higher in the Len+DEB-TACE+HAIC group (Fig. 2) than the Len+DEB-TACE group. The DCR of overall tumor (90.6% vs. 77.6%, *P* = 0.021), intrahepatic tumor (94.1% vs. 81.2%, *P* = 0.010) and PVTT (95.3% vs. 85.9%, *P* = 0.036) was also higher in the Len+DEB-TACE+HAIC group than the Len+DEB-TACE group.

**Table 2:** Tumor response for the patients in matched cohorts.

Response	Len+DEB-TACE+HAIC group (*n* = 85)	Len+DEB-TACE group (*n* = 85)	*P*
Overall tumor			
CR, *n* (%)	6 (7.1)	4 (4.7)	
PR, *n* (%)	46 (54.1)	25 (29.4)	
SD, *n* (%)	25 (29.4)	37 (43.5)	
PD, *n* (%)	8 (9.4)	19 (22.4)	
ORR, %	61.2	34.1	< 0.001
DCR, %	90.6	77.6	0.021
Intrahepatic tumor			
CR, *n* (%)	11 (12.9)	6 (7.1)	
PR, *n* (%)	44 (51.8)	25 (29.4)	
SD, *n* (%)	25 (29.4)	38 (44.7)	
PD, *n* (%)	5 (5.9)	16 (18.8)	
ORR, %	64.7	36.5	< 0.001
DCR, %	94.1	81.2	0.010
PVTT			
CR, *n* (%)	26 (30.6)	9 (10.6)	
PR, *n* (%)	37 (43.5)	31 (36.5)	
SD, *n* (%)	18 (21.2)	34 (40.0)	
PD, *n* (%)	4 (4.7)	11 (12.9)	
ORR, %	74.1	47.1	< 0.001
DCR, %	95.3	85.9	0.036

CR, complete response; DCR, disease control rate; Len+DEB-TACE+HAIC, lenvatinib plus drug-eluting bead transarterial chemoembolization and hepatic arterial infusion chemotherapy; Len+DEB-TACE, lenvatinib plus drug-eluting bead transarterial chemoembolization; ORR, objective response rate; PD, progressive disease; PR, partial response; PVTT, portal vein tumor thrombosis; SD, stable disease.

**Figure 3:**
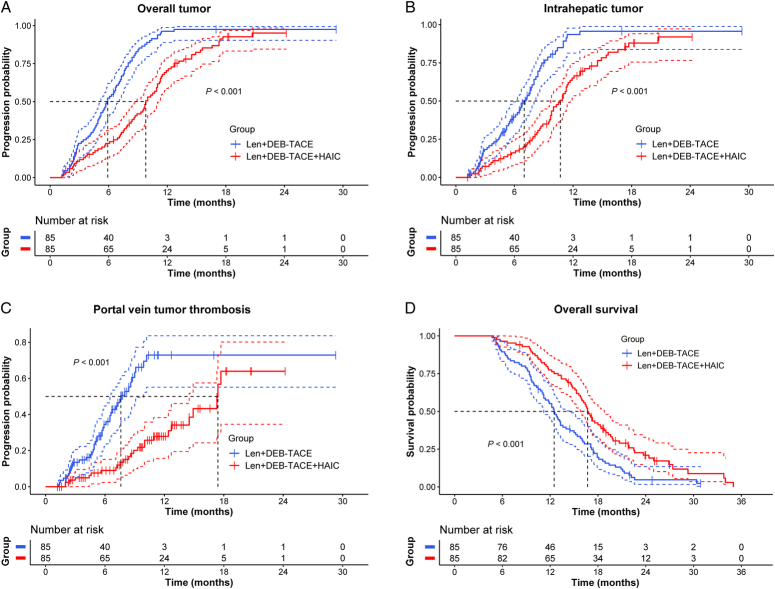
Kaplan–Meier curves for time to progression (TTP) and overall survival (OS) of the patients in matched cohorts according to treatment modality. (A) TTP of overall tumor. (B) TTP of intrahepatic tumor. (C) TTP of portal vein tumor thrombosis. (D) OS. Len+DEB-TACE, lenvatinib plus drug-eluting bead transarterial chemoembolization; Len+DEB-TACE+HAIC, lenvatinib plus drug-eluting bead transarterial chemoembolization and hepatic arterial infusion chemotherapy.

**Figure 4:**
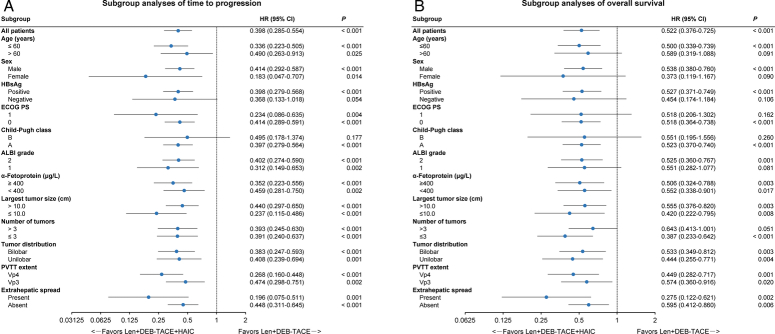
Subgroup analyses of time to progression (A) and overall survival (B). ALBI, albumin-bilirubin; ECOG PS, Eastern Cooperative Oncology Group performance status; HBsAg, hepatitis B surface antigen; HR, hazard ratio; Len+DEB-TACE, lenvatinib plus drug-eluting bead transarterial chemoembolization; Len+DEB-TACE+HAIC, lenvatinib plus drug-eluting bead transarterial chemoembolization and hepatic arterial infusion chemotherapy; PVTT, portal vein tumor thrombosis.

During follow-up, 75 patients (88.2%) in the Len+DEB-TACE+HAIC group and 81 patients (95.3%) in the Len+DEB-TACE group experienced disease progression. The median TTP of overall tumor, intrahepatic tumor and PVTT were 9.8 (95% CI: 8.7–11.2) months, 10.7 (95% CI: 9.7–11.7) months and 17.4 (95% CI: 14.5–not reached) months, respectively, in the Len+DEB-TACE+HAIC group, and 5.9 (95% CI: 5.2–7.0) months, 7.0 (95% CI: 6.1–8.0) months and 7.6 (95% CI: 6.5–9.9) months, respectively, in the Len+DEB-TACE group (all *P* < 0.001; Fig. 3A-C). By the end of follow-up, 71 patients (83.5%) in the Len+DEB-TACE+HAIC group and 79 patients (92.9%) in the Len+DEB-TACE group had died. The median OS was 16.7 (95% CI: 15.8–18.8) months in the Len+DEB-TACE+HAIC group and 12.5 (95% CI: 11.2–14.5) months in the Len+DEB-TACE group (*P* < 0.001; Fig. 3D). Multivariate analyses identified that treatment with Len+DEB-TACE+HAIC was an independent protective factor for TTP and OS (Supplementary Table 1, Supplemental Digital Content 2, http://links.lww.com/JS9/C744). Sensitivity analyses (Supplementary Materials, Supplemental Digital Content 2, http://links.lww.com/JS9/C744) confirmed these findings. Subgroup analyses showed that trends of lower risk of disease progression and death were achieved with Len+DEB-TACE+HAIC over Len+DEB-TACE in almost all the subgroups (Fig. 4A, B).

### Safety

In the study, all TRAEs were not unexpected, and there was no treatment-related death. The overall incidence of TRAEs was similar between the two groups (Table 3). Decreased platelet (36.5% vs. 22.4%, *P* = 0.043), decreased leukocyte (34.1% vs. 18.8%, *P* = 0.024), nausea/vomiting (35.3% vs. 21.2%, *P* = 0.041), decreased appetite (28.2% vs. 12.9%, *P* = 0.014), and sensory neuropathy (9.4% vs. 0%, *P* = 0.011) occurred more frequently in the Len+DEB-TACE+HAIC group than the Len+DEB-TACE group. However, the incidences of all grade 3/4 TRAEs were not significantly different between the two groups.

**Table 3:** Treatment-related adverse events in the matched cohorts.

	Any grade			Grade 3–4		
Adverse events	Len+DEB-TACE+HAIC group (*n* = 85)	Len+DEB-TACE group (*n* = 85)	*P*	Len+DEB-TACE+HAIC group (*n* = 85)	Len+DEB-TACE group (*n* = 85)	*P*
Total, *n* (%)	82 (96.5)	81 (95.3)	> 0.999	33 (38.8)	29 (34.1)	0.524
Abdominal pain, *n* (%)	56 (65.9)	50 (58.8)	0.342	11 (12.9)	14 (16.5)	0.516
Fever, *n* (%)	44 (51.8)	48 (56.5)	0.538	4 (4.7)	6 (7.1)	0.514
Decreased platelet, *n* (%)	31 (36.5)	19 (22.4)	0.043	9 (10.6)	3 (3.5)	0.072
Hypertension, *n* (%)	30 (35.3)	33 (38.8)	0.634	18 (21.2)	15 (17.6)	0.561
Diarrhea, *n* (%)	30 (35.3)	24 (28.2)	0.323	2 (2.4)	1 (1.2)	> 0.999
Nausea/vomiting, *n* (%)	30 (35.3)	18 (21.2)	0.041	6 (7.1)	3 (3.5)	0.493
Weight loss, *n* (%)	29 (34.1)	21 (24.7)	0.178	2 (2.4)	1 (1.2)	> 0.999
Decreased leukocyte, *n* (%)	29 (34.1)	16 (18.8)	0.024	7 (8.2)	2 (2.4)	0.171
Decreased appetite, *n* (%)	24 (28.2)	11 (12.9)	0.014	2 (2.4)	1 (1.2)	> 0.999
Elevated ALT, *n* (%)	23 (27.1)	20 (23.5)	0.597	5 (5.9)	7 (8.2)	0.549
Elevated AST, *n* (%)	21 (24.7)	20 (23.5)	0.858	3 (3.5)	4 (4.7)	> 0.999
Decreased hemoglobin, *n* (%)	21 (24.7)	13 (15.3)	0.125	6 (7.1)	2 (2.4)	0.277
Fatigue, *n* (%)	20 (23.5)	14 (16.5)	0.250	4 (4.7)	2 (2.4)	0.678
Hand-foot syndrome, *n* (%)	16 (18.8)	15 (17.6)	0.843	0 (0)	0	—
Proteinuria, *n* (%)	15 (17.6)	14 (16.5)	0.838	2 (2.4)	3 (3.5)	> 0.999
Hypothyroidism, *n* (%)	15 (17.6)	18 (21.2)	0.561	0 (0)	0	—
Elevated bilirubin, *n* (%)	11 (12.9)	13 (15.3)	0.660	5 (5.9)	3 (3.5)	0.717
Hypoalbuminemia, *n* (%)	9 (10.6)	13 (15.3)	0.361	2 (2.4)	3 (3.5)	> 0.999
Ascites/pleural effusion *n* (%)	8 (9.4)	9 (10.6)	0.798	1 (1.2)	2 (2.4)	> 0.999
Sensory neuropathy, *n* (%)	8 (9.4)	0	0.011	0	0	—
Inguinal hematoma, *n* (%)	6 (7.1)	4 (4.7)	0.514	0	0	—
Bile duct dilation/biloma, *n* (%)^a^	5 (5.9)	8 (9.4)	0.387	1 (1.2)	2 (2.4)	> 0.999
Rash, *n* (%)	4 (4.7)	3 (3.5)	> 0.999	0	1 (1.2)	> 0.999
Cholecystitis, *n* (%)	3 (3.5)	3 (3.5)	> 0.999	2 (2.4)	1 (1.2)	> 0.999
Constipation, *n* (%)	2 (2.4)	3 (3.5)	> 0.999	0 (0)	0	—
Gastrointestinal hemorrhage, *n* (%)^b^	2 (2.4)	2 (2.4)	> 0.999	2 (2.4)	2 (2.4)	> 0.999
Liver abscess, *n* (%)^c^	1 (1.2)	2 (2.4)	> 0.999	1 (1.2)	2 (2.4)	> 0.999

Data are numbers of patients, with percentages in parentheses.

^a^ One patient in the Len+DEB-TACE+HAIC group and two patients in the Len+DEB-TACE group developed biloma (grade 3), which was considered to be related to DEB-TACE and successfully managed by percutaneous transhepatic biloma drainage.

^b^ Two patients in each group suffered from variceal bleeding (grade 4) after DEB-TACE+HAIC/DEB-TACE, which was managed by endoscopic treatment and/or transjugular intrahepatic portosystemic shunt.

^c^ One patient in the Len+DEB-TACE+HAIC group and two patients in the Len+DEB-TACE group developed liver abscess (grade 3), which was considered to be related to DEB-TACE and successfully managed by percutaneous transhepatic abscess drainage.

ALT, alanine aminotransferase; AST, aspartate aminotransferase; DEB-TACE, drug-eluting bead transarterial chemoembolization; Len+DEB-TACE, lenvatinib plus drug-eluting bead transarterial chemoembolization; Len+DEB-TACE+HAIC, lenvatinib plus drug-eluting bead transarterial chemoembolization and hepatic arterial infusion chemotherapy.

TRAEs led to interruption, dose reduction and discontinuation of lenvatinib in 52 (61.2%), 50 (58.8%) and 8 (9.4%) patients, respectively, in the Len+DEB-TACE+HAIC group, and in 46 (54.1%), 45 (52.9%) and 7 (8.2%) patients, respectively, in the Len+DEB-TACE group. TRAEs led to delay, dose reduction and discontinuation of DEB-TACE+HAIC in 18 (21.2%), 35 (41.2%) and 23 (27.1%) patients, respectively, in the Len+DEB-TACE+HAIC group. TRAEs led to delay and discontinuation of DEB-TACE in 13 (15.3%) and 6 (7.1%) patients, respectively, in the Len+DEB-TACE group. There were six patients (7.1%) in the Len+DEB-TACE+HAIC group and four patients (4.7%) in the Len+DEB-TACE group with discontinuation of both lenvatinib and DEB-TACE+HAIC/DEB-TACE because of TRAEs.

## Discussion

This multicenter, retrospective, matched cohort study revealed that Len+DEB-TACE+HAIC was associated with better ORR, DCR, TTP, and OS over Len+DEB-TACE for patients with HCC greater than 7 cm and major PVTT. These findings were consistently substantiated by all sensitivity and subgroup analyses for TTP and OS. Additionally, the overall incidence of TRAEs in the Len+DEB-TACE+HAIC group was similar to that in the Len+DEB-TACE group. All these results suggested that, compared with Len+DEB-TACE, Len+DEB-TACE+HAIC might be a superior therapeutic strategy for large HCC with major PVTT.

Previous studies^16,32,33^, including the LAUNCH trial, have demonstrated that lenvatinib plus TACE could provide better survivals over lenvatinib alone in unresectable/advanced HCC, and suggested that tumor debulking by TACE might increase the efficacy of lenvatinib. In our study, with Len+DEB-TACE+HAIC, tumor responses and TTP of overall tumor, intrahepatic tumor and PVTT were significantly improved, while the number of transarterial treatments was less in the Len+DEB-TACE+HAIC group than the Len+DEB-TACE group. These results indicated that adding HAIC to Len+DEB-TACE was more effective in controlling tumors and impeding disease progression, which further supported the hypothesis that tumor debulking through a potent locoregional treatment might enhance the efficacy of lenvatinib, thereby prolonging survival.

The reasons for the clinical benefits achieved with Len+DEB-TACE+HAIC might be as follows: (1) DEB-TACE caused extensive tumor necrosis but inevitably led to hypoxia-induced angiogenesis, which might be suppressed by the antiangiogenic agent lenvatinib^15^. (2) Lenvatinib might increase chemotherapeutic drug delivery by inhibiting tumor angiogenesis and promoting vascular normalization^17^. (3) Since patients included in this study all have large tumor burden, for reducing the risk of DEB-TACE complications, complete tumor embolization was planned to be achieved by greater than or equal to 2 DEB-TACE sessions. In this case, HAIC exposed the residual tumors on DEB-TACE to high-dose chemotherapeutic drugs^9,17^, while embolization could reduce the risk of chemotherapy resistance^34^, which together led to better tumor control without the need for an excessive embolization per DEB-TACE. Additionally, the procedures of DEB-TACE and HAIC we adopted might partly contributed to the favorable outcomes. First, although there was no sufficient evidence that DEB-TACE was superior to conventional TACE for HCC^5,35^, DEB-TACE was still often performed for patients with large tumor burden in the participating centers. It has been reported that DEB-TACE was associated with higher tumor response and better tolerance for patients with more advanced disease or large tumor burden^5,36,37^. Second, large HCCs often have extrahepatic collateral arteries^17^. In case of extrahepatic tumor-feeding arteries they were first chemoembolized before hepatic artery catheterization for chemoembolization or infusion chemotherapy. In this way, the tumor-feeding arteries could be treated as much as possible, thus providing a better tumor control.

Interestingly, although the patients included in our study all had major PVTT, an ORR of 74.1% and a median TTP of 17.4 months for PVTT were achieved with the treatment of Len+DEB-TACE+HAIC, which were significantly better than those with Len+DEB-TACE, indicating that the addition of HAIC to Len+DEB-TACE also had significant activity in controlling PVTT. We thought that the effectiveness of HAIC and DEB-TACE for PVTT was based on the fact that PVTT, like intrahepatic tumors, mainly received blood supply through hepatic arteries^38^. Additionally, in our study, for the patients with arterioportal shunts, with the combination of DEB-TACE, the shunts were embolized before arterial infusion of chemotherapy drugs, which might prevent drug loss through the shunts and increase the dose of drugs for tumors. Together, our findings suggested that Len+DEB-TACE+HAIC had substantial therapeutic effects on intrahepatic tumor and PVTT, both of which contributed to the improved TTP and survival of patients with large HCC and major PVTT.

Previous studies^16,32,33,39^ have reported an ORR of 46.5–57.5%, a TTP/progression-free survival of 4.7–10.6 months, and an OS of 13.8–17.8 months for lenvatinib plus TACE in unresectable HCC patients, which appeared better than those for Len+DEB-TACE in our study. However, it was noteworthy that all patients included in our study had HCC greater than 7 cm and a considerable proportion of patients had tumor size greater than 10 cm, tumor number greater than 3 or Vp4 PVTT, all of which might result in much poorer prognosis. But in any case, with the addition of HAIC to Len+DEB-TACE, the clinical outcomes of HCC patients with high tumor burden and major PVTT were significantly improved.

In our study, no treatment-related death occurred. All TRAEs in both groups were manageable with appropriate monitoring, treatment interruption, dose modification (for lenvatinib and chemotherapy drugs of HAIC), and/or corresponding interventions. Although more patients in the Len+DEB-TACE+HAIC group experienced any grade decreased platelet, decreased leukocyte, nausea/vomiting, decreased appetite, and sensory neuropathy owing to the cytotoxic agents, no statistically significant differences in grade 3–4 TRAEs between the two groups were observed. These results suggested an acceptable safety profile of Len+DEB-TACE+HAIC.

Our study had some limitations. First, the retrospective nature of this study and the treatment preferences inevitably led to selection bias. To reduce the potential effects of the bias on evaluation of outcomes, the PSM analysis and several sensitivity analyses were applied. Second, although standardized TACE were carried out in the participating centers, the quality and completeness of DEB-TACE and HAIC were difficult to guarantee, and their potential effects on clinical outcomes remained to be concerned. Additionally, the sample size of our study was limited. It is necessary to verify our findings with large-scale randomized controlled trials.

In conclusion, our study showed safety and promising therapeutic outcomes with the triple combination of Len+DEB-TACE+HAIC in patients with HCC greater than 7.0 cm and major PVTT. These patients could benefit from Len+DEB-TACE+HAIC with remarkable better tumor response, TTP, and OS compared with Len+DEB-TACE. These findings need to be validated in large sample, randomized controlled trials.

## Ethical approval

This study was approved by the Ethical Committee of the Second Affiliated Hospital of Guangzhou Medical University on October 16, 2023 (approval number: 2023-hg-ks-34).

## Consent

The need for informed consent was waived due to the retrospective nature of the study.

## Source of funding

This work was supported by the National Natural Science Foundation of China (82172043 and 82202273), the Plan on Enhancing Scientific Research in GMU (198), the Innovative and Featured Clinical Technique of Guangzhou, the Basic and Applied Basic Research Foundation of Guangdong Province (2020A1515110654), and the Clinical Study Project of the Second Affiliated Hospital of Guangzhou Medical University (2021-LCYJ-DZX-01, 2022-LCYJ-YY-02, 2022-LCYJ-YYDZX-01, and 2023-LCYJ-ZF-47).

## Author contribution

Conceptualization: M.C., L. Liang, H.C., and K.Z.; Methodology: M.C., L. Liang, and K.Z.; Validation: M.C., L. Liang, J. Zhang., N.C., H.C, and K.Z.; Formal analysis: M.C., L. Liang, and K.Z.; Investigation: all authors; Resources: M.C., J. Zhang., N.C., W.H., X.H., Y.L., C.D., H.D., X.L., H.C., and K.Z.; Data curation: M.C., L. Liang, Y.G., and J. Zhou.; Writing—original draft preparation: M.C., L. Liang, and K.Z.; Writing—review and editing: all authors; Visualization: M.C. and L. Liang; Supervision: H.C. and K.Z.; Project administration: K.Z.; Funding acquisition: M.C., L. Lin, Y.C., and K.Z.; All authors read and approved the final manuscript.

## Conflicts of interest disclosure

The authors declare that they have no conflicts of interest.

## Research registration unique identifying number (UIN)

1. This study is registered at ClinicalTrials.gov, ClinicalTrials. gov ID is NCT06265883.
2. The registration center is: The Second Affiliated Hospital of Guangzhou Medical University.
3. The hyperlink to the registration is: https://clinicaltrials.gov/study/NCT06265883.

## Guarantor

Kangshun Zhu.

## Data availability statement

The datasets used or analyzed during this study are available from the corresponding author upon reasonable request.

## Provenance and peer review

Not commissioned, externally peer-reviewed.

## Supplementary Material

SUPPLEMENTARY MATERIAL
