# Supplementary material for: Lenvatinib plus drug-eluting bead transarterial chemoembolization with/without hepatic arterial infusion chemotherapy for hepatocellular carcinoma larger than 7 cm with major portal vein tumor thrombosis: a multicenter retrospective cohort study
Source: Int J Surg. 2024 Jun 13;110(12):7860–70. doi: 10.1097/JS9.0000000000001819 (PMC11634077; doi:10.1097/JS9.0000000000001819)
Supplement: SUPPLEMENTARY MATERIAL [file js9-110-7860-s002.docx]

**Lenvatinib plus drug-eluting bead transarterial chemoembolization with/without hepatic arterial infusion chemotherapy for hepatocellular carcinoma larger than 7 cm with major portal vein tumor thrombosis: a multicenter retrospective cohort study**

**Supplementary Materials**

***Detailed DEB-TACE and HAIC procedures***

The DEB-TACE procedures were carried out by physicians with more than 10 years of experience in interventional radiology from the participating centers according to standardization protocols^[1,2]^. Before starting the procedure, the DEBs were prepared by mixing a vial of 100-300 μm CalliSpheres (Hengrui Medical) or DC Bead (Biocompatibles) with a solution of 40-60 mg pirarubicin or epirubicin. At the initiation of DEB-TACE, selective arteriography was performed to assess the vascular anatomy and tumor vascularity. During chemoembolization, the DEBs mixed with contrast medium were administered into the tumor-feeding vessels via superselective catheterization^[3-5]^. If blushed tumors were still visible after embolization with a vial of the beads, regular microspheres (8Spheres, Hengrui Medical; Embosphere, Biosphere Medical) with diameters of 100-700 μm were introduced. In order to reduce the risk of complications, the embolization endpoint with blood stasis of the tumor-feeding arteries was not achieved in a single treatment but in 2-3 DEB-TACE sessions^[4-6]^. If the tumors had extrahepatic feeding arteries (inferior phrenic artery, intercostal artery, or internal mammary artery, etc.), these arteries were chemoembolized first before cannulation of the hepatic feeding arteries. In the case of arterioportal shunting, the shunts were embolized with polyvinyl alcohol particles (90-500 μm; Cook) before injection of DEBs.

For HAIC combination therapy, the microcatheter was not removed but reserved in the main tumor-feeding hepatic artery after chemoembolization^[5]^. Afterwards, the patients were transferred to the ward for drug infusion via the microcatheter: oxaliplatin, 85 mg/m^2^ for 2 hours; leucovorin, 400 mg/m^2^ for 2 hours; and flurouracil, 400 mg/m^2^ bolus and 2400 mg/m^2^ for 46 hours.

Subsequent DEB-TACE or DEB-TACE+HAIC was repeated for viable tumors demonstrated by follow-up imaging in patients without worsening liver function or contraindications^[1]^. In the case of clinical or functional deterioration, DEB-TACE and/or HAIC could be delayed and dose reduction of chemotherapy agents for HAIC was allowed^[7,8]^. In the Len+DEB-TACE+HAIC group, DEB-TACE or HAIC could be performed alone when the other one was discontinued due to adverse events or technical difficulties. In both groups, the transarterial treatment (i.e., DEB-TACE and/or HAIC) was discontinued if objective response was not achieved after 2-3 consecutive treatment sessions.

***Assessment of PVTT response***

The treatment response of PVTT was categorized by using a modified standard as described in our previous study^[9]^: complete response, complete disappearance or shrinkage of the PVTT, or complete disappearance of enhancement inside the PVTT; partial response, greater than or equal to 30% decrease in the largest perpendicular diameter of the PVTT, or PVTT shrinking back to a higher-order branch of portal vein; progressive disease, greater than or equal to 20% increase in the largest perpendicular diameter of the PVTT, or PVTT extending to a more proximal portal vein; stable disease, a response between partial response and progressive disease.

***Sensitivity analyses***

Three sensitivity analyses were conducted to assess the robustness of the PSM analysis. First, a 1:1 PSM analysis was performed using optimal matching method without replacement. Propensity scores were generated using a logistic regression model with variables of age, sex (male/female), HBsAg (positive/negative), ECOG PS (1/0), Child-Pugh class (B/A), ALBI grade (2/1), α-fetoprotein, largest tumor size, number of tumors (> 3/≤ 3), tumor distribution (bilobar/unilobar), PVTT extent (Vp4/Vp3) and extrahepatic spread (present/absent). After matching, 100 patients remained in each group. There were still some baseline characteristics which were not well balanced between the two groups. The ORR of overall tumor (64.0% vs. 36.0%, *P* < 0.001), intrahepatic tumor (67.0% vs. 38.0%, *P* < 0.001) and PVTT (74.0% vs. 48.0%, *P* < 0.001) was higher in the Len+DEB-TACE+HAIC group than the Len+DEB-TACE group. The DCR of overall tumor (92.0% vs. 78.0%, *P* = 0.006), intrahepatic tumor (95.0% vs. 82.0%, *P* = 0.004) and PVTT (96.0% vs. 86.0%, *P* = 0.013) was also higher in the Len+DEB-TACE+HAIC group than the Len+DEB-TACE group. The median TTP of overall tumor, intrahepatic tumor and PVTT were 9.8 (95% CI: 8.6-11.0) months, 10.7 (95% CI: 9.7-11.6) months and 17.4 (95% CI: 14.9-not reached) months, respectively, in the Len+DEB-TACE+HAIC group, and 5.9 (95% CI: 5.3-7.2) months, 7.3 (95% CI: 6.3-8.4) months and 8.3 (95% CI: 6.7-10.0) months, respectively, in the Len+DEB-TACE group (all *P* < 0.001). The median OS was 16.8 (95% CI: 15.8-19.0) months in the Len+DEB-TACE+HAIC group and 12.6 (95% CI: 11.4-14.5) months in the Len+DEB-TACE group (*P* < 0.001).

Second, a 1:1 PSM analysis was performed using nearest-neighbor method and a caliper width of 0.2 standard deviation without replacement. Propensity scores were generated using a logistic regression model with several key clinical variables (ECOG PS, Child-Pugh class, ALBI grade, largest tumor size, number of tumors, tumor distribution, PVTT extent and extrahepatic spread). The excluded variables (age, sex, HBsAg, α-fetoprotein) were either balanced before matching or not definitive prognostic for TTP or OS in previous studies. After matching, 83 patients remained in each group. Some baseline characteristics were not well balanced between the two groups. The ORR of overall tumor (67.5% vs. 34.9%, *P* < 0.001), intrahepatic tumor (71.1% vs. 37.3%, *P* < 0.001) and PVTT (75.9% vs. 47.0%, *P* < 0.001) was higher in the Len+DEB-TACE+HAIC group than the Len+DEB-TACE group. The DCR of overall tumor (91.6% vs. 77.1%, *P* = 0.010), intrahepatic tumor (95.2% vs. 81.9%, *P* = 0.007) and PVTT (96.4% vs. 85.5%, *P* = 0.015) was also higher in the Len+DEB-TACE+HAIC group than the Len+DEB-TACE group. The median TTP of overall tumor, intrahepatic tumor and PVTT were 10.0 (95% CI: 9.3-11.3) months, 10.7 (95% CI: 9.8-11.7) months and not reached (95% CI: 14.9-not reached), respectively, in the Len+DEB-TACE+HAIC group, and 6.3 (95% CI: 5.4-7.3) months, 7.6 (95% CI: 6.7-8.5) months and 8.4 (95% CI: 7.1-10.2) months, respectively, in the Len+DEB-TACE group (all *P* < 0.001). The median OS was 17.1 (95% CI: 15.8-19.8) months in the Len+DEB-TACE+HAIC group and 12.8 (95% CI: 11.4-15.0) months in the Len+DEB-TACE group (*P* < 0.001).

For the inverse probability of treatment weighting analysis, the propensity score (probability of receiving Len+DEB-TACE+HAIC) for each patient was generated by using a logistic regression model with variables of age, sex, HBsAg, ECOG PS, Child-Pugh class, ALBI grade, α-fetoprotein, largest tumor size, number of tumors, tumor distribution, PVTT extent and extrahepatic spread. The individual weights were then calculated using the propensity scores as follows: 1/propensity score for patients receiving Len+DEB-TACE+HAIC, and 1/(1-propensity score) for patients receiving Len+DEB-TACE. The median TTP of overall tumor, intrahepatic tumor and PVTT were 10.0 (95% CI: 8.7-11.3) months, 10.9 (95% CI: 9.8-11.7) months and not reached (95% CI: 14.9-not reached), respectively, in the Len+DEB-TACE+HAIC group, and 5.9 (95% CI: 5.2-7.0) months, 7.2 (95% CI: 6.3-8.2) months and 8.4 (95% CI: 7.1-10.2) months, respectively, in the Len+DEB-TACE group (all *P* < 0.001). The median OS was 17.1 (95% CI: 15.9-19.3) months in the Len+DEB-TACE+HAIC group and 12.5 (95% CI: 11.2-14.5) months in the Len+DEB-TACE group (*P* < 0.001).

**References**

[1] Zhou J, Sun HC, Wang Z, *et al*. Guidelines for Diagnosis and Treatment of Primary Liver Cancer in China (2017 Edition). Liver Cancer 2018;7:235-60.

[2] Chinese College of Interventionalists, Chinese Medical Doctor Association. Chinese Clinical Practice Guidelines for transarterial chemoembolization of hepatocellular carcinoma. Zhonghua Gan Zang Bing Za Zhi 2019;27:172-81.

[3] Liu Y, Huang W, He M, *et al*. Efficacy and Safety of CalliSpheres Drug-Eluting Beads Transarterial Chemoembolization in Barcelona Clinic Liver Cancer Stage C Patients. Oncol Res 2019;27:565-73.

[4] Cai M, Huang W, Huang J, *et al*. Transarterial Chemoembolization Combined With Lenvatinib Plus PD-1 Inhibitor for Advanced Hepatocellular Carcinoma: A Retrospective Cohort Study. Front Immunol 2022;13:848387.

[5] Huang J, Huang W, Zhan M, *et al*. Drug-Eluting Bead Transarterial Chemoembolization Combined with FOLFOX-Based Hepatic Arterial Infusion Chemotherapy for Large or Huge Hepatocellular Carcinoma. J Hepatocell Carcinoma 2021;8:1445-58.

[6] Lencioni R, de Baere T, Burrel M, *et al*. Transcatheter treatment of hepatocellular carcinoma with Doxorubicin-loaded DC Bead (DEBDOX): technical recommendations. Cardiovasc Intervent Radiol 2012;35:980-5.

[7] He M, Li Q, Zou R, *et al*. Sorafenib Plus Hepatic Arterial Infusion of Oxaliplatin, Fluorouracil, and Leucovorin vs Sorafenib Alone for Hepatocellular Carcinoma With Portal Vein Invasion: A Randomized Clinical Trial. Jama Oncol 2019;5:953-60.

[8] Liu BJ, Gao S, Zhu X, *et al*. Sorafenib combined with embolization plus hepatic arterial infusion chemotherapy for inoperable hepatocellular carcinoma. World J Gastrointest Oncol 2020;12:663-76.

[9] Guo Y, Wu J, Liang L, *et al*. Tyrosine-kinase inhibitor combined with iodine-125 seed brachytherapy for hepatocellular carcinoma refractory to transarterial chemoembolization: a propensity-matched study. Cancer Imaging 2023;23:91.

**Supplementary Table 1**

**Analyses of prognostic factors for time to progression and overall survival in the matched cohorts**

| **Factor** | **Univariate analysis** | |  | **Multivariate analysis** | |
| --- | --- | --- | --- | --- | --- |
|  | **HR (95% CI)** | ***P*** |  | **HR (95% CI)** | ***P*** |
| TTP analyses |  |  |  |  |  |
| Treatment (Len+DEB-TACE+HAIC) | 0.398 (0.285-0.554) | < 0.001 |  | 0.364 (0.259-0.512) | < 0.001 |
| Age (≤ 60 years) | 1.326 (0.930-1.889) | 0.119 |  |  |  |
| Sex (male) | 1.000 (0.585-1.708) | 0.999 |  |  |  |
| HBsAg (positive) | 0.778 (0.485-1.247) | 0.297 |  |  |  |
| ECOG PS (1) | 1.380 (0.868-2.193) | 0.173 |  |  |  |
| Child-Pugh class (B) | 1.078 (0.650-1.787) | 0.772 |  |  |  |
| ALBI grade (2) | 1.156 (0.803-1.664) | 0.435 |  |  |  |
| α-Fetoprotein (≥ 400 μg/L) | 0.974 (0.707-1.342) | 0.874 |  |  |  |
| Largest tumor size (> 10.0 cm) | 1.044 (0.737-1.478) | 0.808 |  |  |  |
| Number of tumors (> 3) | 1.309 (0.950-1.804) | 0.100 |  |  | 0.310 |
| Tumor distribution (bilobar) | 1.258 (0.908-1.742) | 0.168 |  |  |  |
| PVTT extent (Vp4 ) | 1.445 (1.050-1.987) | 0.024 |  | 1.672 (1.206-2.318) | 0.002 |
| Extrahepatic spread (present) | 1.019 (0.687-1.513) | 0.924 |  |  |  |
| OS analyses |  |  |  |  |  |
| Treatment (Len+DEB-TACE+HAIC) | 0.522 (0.376-0.725) | < 0.001 |  | 0.501 (0.360-0.697) | < 0.001 |
| Age (≤ 60 years) | 1.059 (0.742-1.511) | 0.751 |  |  |  |
| Sex (male) | 1.165 (0.671-2.023) | 0.588 |  |  |  |
| HBsAg (positive) | 1.160 (0.705-1.909) | 0.559 |  |  |  |
| ECOG PS (1) | 1.445 (0.897-2.326) | 0.130 |  |  |  |
| Child-Pugh class (B) | 1.277 (0.741-2.198) | 0.378 |  |  |  |
| ALBI grade (2) | 1.230 (0.847-1.788) | 0.277 |  |  |  |
| α-Fetoprotein (≥ 400 μg/L) | 1.160 (0.839-1.604) | 0.369 |  |  |  |
| Largest tumor size (> 10.0 cm) | 1.076 (0.757-1.529) | 0.682 |  |  |  |
| Number of tumors (> 3) | 1.305 (0.936-1.818) | 0.116 |  |  |  |
| Tumor distribution (bilobar) | 1.264 (0.900-1.776) | 0.177 |  |  |  |
| PVTT extent (Vp4 ) | 1.496 (1.077-2.076) | 0.016 |  | 1.591 (1.143-2.214) | 0.006 |
| Extrahepatic spread (present) | 1.001 (0.675-1.484) | 0.997 |  |  |  |

Analyses were performed using Cox proportional hazard regression model.

ALBI, albumin-bilirubin; CI, confidence interval; ECOG PS, Eastern Cooperative Oncology Group performance status; HBsAg, hepatitis B surface antigen; HR, hazard ratio; Len+DEB-TACE+HAIC, lenvatinib plus drug-eluting bead transarterial chemoembolization and hepatic arterial infusion chemotherapy; OS, overall survival; PVTT, portal vein tumor thrombosis; TTP, time to progression.

**Supplementary Figure 1**


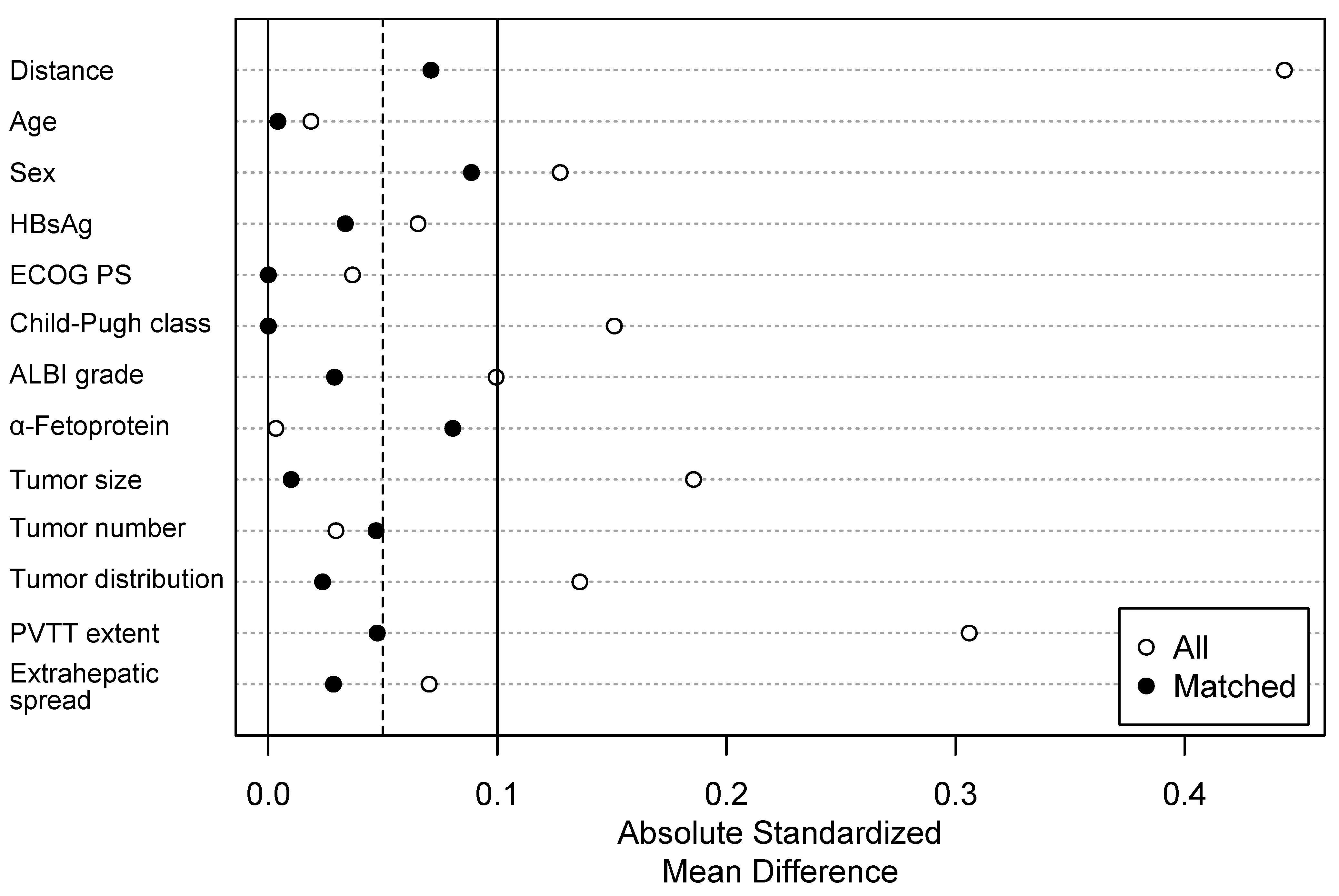


**Supplementary Figure 1.** Absolute standardized mean difference of propensity score matching. ALBI, albumin-bilirubin; ECOG PS, Eastern Cooperative Oncology Group performance status; HBsAg, Hepatitis B surface antigen; PVTT, portal vein tumor thrombosis.
